# Five New Species of *Gibellula* (Hypocreales, Cordycipitaceae) from China

**DOI:** 10.3390/jof11120891

**Published:** 2025-12-17

**Authors:** Bo Tu, Hui Chen, Xu Zhang, Yu-Hu Guan, De-Xiang Tang, Qi-Rui Li, Yao Wang

**Affiliations:** 1 State Key Laboratory of Functions and Applications of Medicinal Plants, Guizhou Medical University, Guian New District, Guiyang 561113, China; tb3318@gmc.edu.cn; 2 State Key Laboratory of Discovery and Utilization of Functional Components in Traditional Chinese Medicine & School of Pharmaceutical Sciences, Guizhou Medical University, Guian New District, Guiyang 561113, China; chui68769@gmail.com (H.C.); xuzhanggmu2018@163.com (X.Z.); 15085720191@163.com (Y.-H.G.); tangdx1516@163.com (D.-X.T.); lqrnd2008@163.com (Q.-R.L.); 3 The High Efficacy Application of Natural Medicinal Resources Engineering Center of Guizhou Province, Guizhou Medical University, Guian New District, Guiyang 561113, China

**Keywords:** new species, spider-pathogenic fungi, *Gibellula*, taxonomy, phylogenetics

## Abstract

The genus *Gibellula* (Cordycipitaceae, Hypocreales) comprises highly specialized, obligate pathogens that exclusively parasitize spiders. In this study, five new species were delimited based on morphological and phylogenetic evidence from a six-locus dataset (nr*SSU*, ITS, nr*LSU*, *tef-1α*, *rpb1*, *rpb2*). Specimens were collected from northeastern (Jilin and Liaoning Provinces) and southwestern (Yunnan Province) China. Phylogenetic analyses resolved these collections into five distinct, well-supported lineages, described as *G. baishanensis*, *G. jilinensis*, *G. kunmingensis*, *G. paralongispora*, and *G. yunnanensis* spp. nov. Among these, *G. baishanensis* and *G. jilinensis* were identified as sister taxa, whereas *G. kunmingensis* formed an independent lineage. *Gibellula paralongispora* was recovered as a sister to *G. longispora*, and *G. yunnanensis* as a sister to *G. attenboroughii*; both new species are supported by significant morphological distinctions (e.g., conidiophore length and conidial shape). This study provides detailed descriptions, illustrations, and morphological comparisons for these taxa, thereby enriching the taxonomy of *Gibellula*. Furthermore, the records from Jilin and Liaoning represent only the second documented occurrence of the genus in northeastern China, significantly expanding its known geographic range.

## 1. Introduction

The order Hypocreales (Sordariomycetes, Ascomycota) comprises a diverse group of fungi, many of which are entomopathogens known to infect insects and spiders [1,2]. Among these, the families Clavicipitaceae, Cordycipitaceae, Ophiocordycipitaceae, and Polycephalomycetaceae represent the most species-rich lineages of arthropod-pathogenic fungi [3,4,5,6,7]. To date, approximately 90 fungal species have been reported as pathogens of spiders (Araneae), spanning 16 families [8]. These arachnopathogenic fungi are distributed across 16 genera, including *Arachnidicola*, *Beauveria*, *Clonostachys*, *Cordyceps*, *Engyodontium*, *Gibellula*, *Hevansia*, *Hirsutella*, *Hymenostilbe*, *Jenniferia*, *Lecanicillium*, *Ophiocordyceps*, *Parahevansia*, *Polystromomyces*, *Purpureocillium*, and *Torrubiella* [9,10,11,12]. Of particular interest is the genus *Gibellula*, which is distinguished by its exclusive pathogenicity to spiders and its high degree of host specificity.

The genus *Gibellula* was established by Cavara in 1894, with *G. pulchra* designated as the type species [13]. This taxon is characterized by aspergillus-like conidiophores terminating in a vesicle that bears metulae, each supporting clusters of phialides which, in turn, produce chains of conidia. Despite its distinctive morphology, the taxonomic framework of *Gibellula* remains partially unresolved, primarily due to the absence of a clearly designated type specimen and viable cultures of *G. pulchra*, leading to persistent nomenclatural ambiguity [10]. Phylogenetic studies currently recognize 23 species, although molecular data are still lacking for several taxa. Sexual–asexual connections have been confirmed for only nine species; the sexual morphs typically form superficial, ovoid perithecia on loose mycelial mats, with cylindrical asci featuring a thickened apex and filiform ascospores that disarticulate into part-spores [10,14,15,16,17,18]. Most species, however, are known exclusively from their asexual morphs, which exhibit either aspergillus-like or penicillium-like conidiophores [10].

*Gibellula* species, commonly found on spiders inhabiting the abaxial surfaces of dicotyledonous leaves, dead stems, and other substrates, exhibit a broad global distribution across temperate, subtropical, and tropical regions, from Argentina and Brazil to China, Japan, the United States, and elsewhere [9,10]. Despite this wide distribution and the documentation of more than ten species in China to date [14,15,16,17,18,19,20], the diversity of *Gibellula* in East Asia remains incompletely explored, particularly in under-surveyed regions. The continual discovery of new taxa through integrated morphological and phylogenetic approaches underscores the need for further investigation.

In this study, we report five spider-pathogenic fungal isolates collected from Jilin, Liaoning and Yunnan Provinces, China. Based on comprehensive morphological examinations and multi-locus phylogenetic analyses, these isolates are proposed as five new species of *Gibellula*. Detailed descriptions and comparisons with morphologically and phylogenetically related taxa are provided.

## 2. Materials and Methods

### 2.1. Specimen Collection and Fungus Isolation

The fungal specimens examined in this study were mainly collected from Jilin Province, with supplementary materials obtained from Yunnan Province, China. During field surveys, specimens were photographed in situ, and relevant ecological data were documented. Samples were transported under controlled conditions (4 °C) in plastic containers and subsequently transferred to the laboratory for identification. Voucher specimens were deposited in the Herbarium of Guizhou Medical University (GMB). For fungal isolation, samples were first subjected to surface sterilization by immersion in 30% hydrogen peroxide for five minutes, followed by two rinses with sterile distilled water. Excess moisture was removed using sterile filter paper [21]. Following aseptic removal of the epidermis, tissue fragments were transferred onto potato dextrose agar (PDA) plates. Purified cultures were obtained by sub-culturing and maintained either on PDA slants at 4 °C for long-term storage or in an incubator at 25 °C for active growth [22]. Living cultures have been deposited in the Guizhou Medical University Culture Collection (GMBC).

### 2.2. Morphological Observations

Morphological characterization was based on asexual reproductive structures developed on the host. Observations were conducted at multiple levels, from macroscopic assessment to detailed examination using dissecting and compound microscopes. Macroscopic characterization focused on the number, colour, shape, and length of synnemata, as well as the colour of the mycelium covering the host. These characteristics were examined using a Nikon SMZ745T stereomicroscope (Tokyo, Japan). Microscopic characterization included assessment of the shape and size of vesicles, metulae, phialides, conidial heads, conidia, and conidiophores, as well as the arrangement pattern of conidiophores on the synnematal surface. Microscopic examination and image capture were performed using a Nikon ECLIPSE Ni compound microscope (Nikon, Tokyo, Japan) equipped with a Canon EOS 700D digital camera. Asexual structures such as phialides and conidia were mounted in lactophenol cotton blue solution for detailed observation. Measurements were conducted using Tarosoft (R) Image Frame Work (v.0.9.7). PDA cultures were studied for important morphological characters such as conidia and phialides.

### 2.3. DNA Extraction, Polymerase Chain Reaction (PCR), and Sequencing

The samples were put in a sterile centrifuge tube and processed until they were completely pulverized using sterile fine rods. The genomic DNA purification kit (Qiagen GmbH, Hilden, Germany) was used to isolate genomic DNA in accordance with the manufacturer’s instructions. The purified DNA was stored at −20 °C. The PCR mixture (25 µL) consisted of 1 µL of DNA template, 1 µL of each forward and reverse primer (10 µM each), 9.5 µL of ddH_2_O, and 12.5 µL of 2× Taq PCR Master Mix (TIANGEN, Beijing, China). The primer combination NS1 and NS4 were used to amplify the nuclear ribosomal small subunit (nr*SSU*) [23]. The primer combination ITS4 and ITS5 were used to amplify the nuclear ribosomal internal transcribed spacer region (ITS) [23]. The primer pair 28F and 28R was used to amplify the nuclear ribosomal large subunit (nr*LSU*) [24]. The primer combination TEF-F and TEF-R were used to amplify the translation elongation factor 1α (*tef-1α*) [25,26]. The primer pairs CRPB1-5′F and CRPB1-5′R, as well as fRPB2-5F and fRPB2-7cR, were used to amplify the largest and second largest subunits of RNA polymerase II (*rpb1* and *rpb2*) [25,27,28]. The PCR assays of the six genes were conducted as described by Wang et al. [29]. An automatic sequence analyzer (BGI Co, Ltd., Shenzhen, China) was used to sequence the PCR products after they had been separated by electrophoresis in 1.0% agarose gels and purified using the Gel Band Purification Kit (Bio Teke Co, Ltd., Beijing, China).

### 2.4. Phylogenetic Analyses

Sequence data for six loci (nr*SSU*, ITS, nr*LSU*, *tef-1α*, *rpb1*, and *rpb2*) were obtained from GenBank; relevant taxonomic information and accession numbers are provided in Table 1. Sequence alignment was performed using MAFFT v.7 (https://mafft.cbrc.jp/alignment/server/ (accessed on 20 October 2025)) and MEGA 7.0.26 [30], with manual adjustments made where necessary. The aligned sequences were concatenated into a single dataset using MEGA 7.0.26. Phylogenetic analyses were conducted using both Maximum Likelihood (ML) and Bayesian Inference (BI) methods. For ML analysis, the GTR + FO + G model was selected as the best-fit model, and branch support was evaluated with 1000 rapid bootstrap replicates. ML analyses were performed using RAxML v.7.0.3 [31], with additional ML analysis carried out in IQ-TREE v.2.1.3, where TIM3 + F + I + G4 was identified as the optimal model based on the Bayesian Information Criterion (BIC), and node support was assessed via ultrafast bootstrapping [32]. For BI, substitution models were selected using jModelTest v.2.1.4 [33]; the GTR + I + G model was applied to nr*SSU*, ITS, nr*LSU*, and *tef-1α* partitions, while GTR + I was used for *rpb1* and *rpb2*. Bayesian analysis was run for 5 million generations in MrBayes v.3.2.7a [34]. *Blackwellomyces kaihuaensis* (HMAS 285455) and *Blackwellomyces lateris* (MFLU 18-0663) were designated as outgroup taxa. Phylogenetic trees were visualized and edited using FigTree v.1.4.4 (http://tree.bio.ed.ac.uk/software/figtree (accessed on 20 October 2025)).

## 3. Results

### 3.1. Sequencing and Phylogenetic Analyses

**Figure 1:**
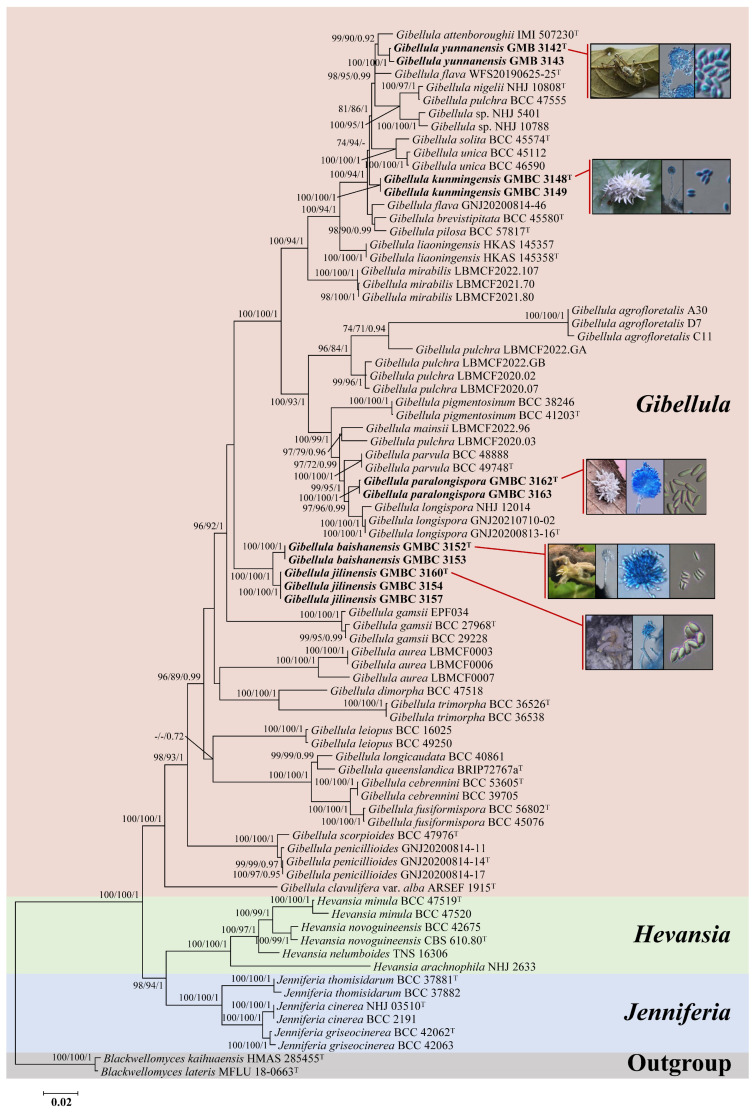
Phylogenetic tree of *Gibellula* and related genera based on a combined six-locus dataset (nr*SSU* + ITS + nr*LSU* + *tef-1a* + *rpb1* + *rpb2*). Branch support values (IQ-TREE-BS/RAxML-BS/BI-PP) above 70%/70%/0.7 are shown. Ex-type materials are marked with “T”. Bold labels indicate sequences generated in this study.

A six-locus dataset (nr*SSU*, ITS, nr*LSU*, *tef-1α*, *rpb1*, and *rpb2*) with a total length of 5696 bp (nr*SSU*: 1076 bp; ITS: 776 bp; nr*LSU*: 949 bp; *tef-1α*: 992 bp; *rpb1*: 776 bp; *rpb2*: 1127 bp) was assembled to elucidate the phylogenetic relationships of *Gibellula* and allied genera in Cordycipitaceae. The alignment included 80 fungal specimens/isolates, comprising 66 of *Gibellula*, six of *Hevansia*, six of *Jenniferia*, and two outgroup specimens of *Blackwellomyces*. Phylogenetic trees reconstructed using maximum likelihood (IQ-TREE, RAxML) and Bayesian inference exhibited highly congruent topologies, with nodal support values (IQ-TREE-BS/RAxML-BS/PP) indicated in Figure 1.

Phylogenetic analyses strongly supported the monophyly of three genera: *Gibellula* (100%/100%/1), *Hevansia* (100%/100%/1), and *Jenniferia* (100%/100%/1). *Hevansia* and *Jenniferia* formed a well-supported sister clade (98%/94%/1), which in turn grouped with *Gibellula* with full support (100%/100%/1). Within the *Gibellula* clade, the five proposed new species each formed well-supported independent lineages: *G. kunmingensis* (GMBC 3148, 3149) as a distinct terminal branch; *G. yunnanensis* (GMB 3142, 3143) forming a sister clade to *G. attenboroughii*; *G. paralongispora* (GMBC 3162, 3163) forming a sister clade to *G. longispora*; and *G. baishanensis* (GMBC 3152, 3153) together with *G. jilinensis* (GMBC 3154, 3157, 3160) forming a distinct clade without a clear sister relationship to any other known *Gibellula* species. The majority of recognized *Gibellula* species were resolved into species-specific clades with high statistical support, further validating the current taxonomic framework.

The combined molecular dataset thus provided robust phylogenetic evidence for the monophyly of *Gibellula*, its phylogenetic position relative to *Hevansia* and *Jenniferia*, and the recognition of five novel species. These results offer a solid foundation for taxonomic revision and evolutionary studies of *Gibellula* and related genera in Cordycipitaceae.

### 3.2. Taxonomy

*Gibellula baishanensis* Y. Wang & H. Chen, sp. nov.

Figure 2.

Mycobank No: 861165

*Etymology*. The specific epithet refers to Baishan City in Jilin Province, China, where the holotype was collected.

*Type*. China, Jilin Province, Baishan City, Fusong County (42.37° N, 127.43° E; alt. 775 m), on a spider on a dead stem, July 2024, Yao Wang (holotype: GMB 3152; ex-type living culture: GMBC 3152).

*Description*. Host surface covered by a dense white mycelial mat. Synnemata white, numerous, arising directly from the entire host body, becoming purplish-grey upon drying. Stipes are short, erect, cylindrical, resembling a narrow head. Conidiophores 69–89 × 8–11 ($\bar{X}$ = 81 × 9, n = 30) μm, few, solitary, white, arising directly from the dorsal surface or legs of the host; erect, thick-walled, septate, with wider spacing between septa toward the apex; simple, with verrucose to globose ornamentation near the apex and conspicuous septal thickening. Terminal cell of conidiophores smooth, thin-walled, 18–29 ($\bar{X}$ = 24, n = 30) μm long, bearing a stipe 6–8 ($\bar{X}$ = 7.3, n = 30) μm long, apically swollen. Vesicles spatulate to conical, 5–11 × 3–5 ($\bar{X}$ = 9.3 × 3.3, n = 30) μm, smooth. Metulae broadly ovoid to broadly ellipsoid, 5–11 × 6–7 ($\bar{X}$ = 8.6 × 6.6, n = 30) μm, borne on vesicles; vesicles together with metulae and phialides forming spherical to ovoid heads, 69–89 × 34.5–44 ($\bar{X}$ = 80 × 39, n = 30) μm. Phialides 10–14 × 2–3 ($\bar{X}$ = 11 × 2.3, n = 30) μm, smooth, tapering, producing conidia in chains. Conidia hyaline, smooth, lacrimoid to subclavate, gradually narrowing toward the base, thin-walled, aseptate, 5.5–7 × 2–4 ($\bar{X}$ = 6 × 3, n = 50) μm. Sexual morph not observed.

*Culture characteristics*. Colonies on PDA grow relatively rapidly at 25 °C, reaching 28–33 mm in diameter after 30 days. Mycelium initially greyish-white to cream-white, with colony margins gradually turning pale yellow. Colonies are loose superficially and compact at the central base. Sporulation not observed in culture.

*Distribution*. China, Jilin Province, Baishan City.

*Other Material Examined*. China, Jilin Province, Baishan City, Fusong County (42.46° N, 127.59° E; alt. 637 m), on a spider on a dead stem, July 2024, Yao Wang (GMB 3153; living culture: GMBC 3153).

*Notes*. Phylogenetically, *G. baishanensis* forms a highly supported clade (IQ-TREE-BS/RAxML-BS/BI-PP = 100%/100%/1) as a sister species to *G. jilinensis*. Morphologically, it is characterized by its large, spherical to ovoid conidial heads (69–89 × 34.5–44 µm) and medium-sized, lacrimoid to subclavate conidia (5.5–7 × 2–4 µm). The size of the conidial heads distinguishes it from species with smaller heads, such as *G. attenboroughii* and *G. agrofloretalis*, while its conidial shape separates it from species with narrowly fusiform conidia and longer conidiophores, such as *G. longispora* and *G. penicillioides*. The combined molecular and morphological evidence supports the recognition of *G. baishanensis* as a distinct species.

*Gibellula jilinensis* Y. Wang & H. Chen, sp. nov.

Figure 3.

Mycobank No: 861166

*Etymology*. The specific epithet refers to Jilin Province, China, where the species was collected.

*Type*. China, Jilin Province, Yanbian Korean Autonomous Prefecture, Dunhua County (43.98° N, 128.31° E; alt. 636 m), on a spider on a dead stem, 26 August 2025, Kun Zhang (holotype: GMB 3160; ex-type living culture: GMBC 3160).

*Description*. Host surface covered by a dense white mycelial mat. Synnemata white, numerous, and arise exclusively from the dorsal surface of the host. During drying, their colour transitions from white to pale yellow, then to deep yellow or brown, and finally to purplish-grey, with spores aggregating on the dorsal surface. Stipes short and stout, cylindrical. Conidiophores few, solitary, yellow to brown, erect, thick-walled, septate, with wider spacing between septa toward the apex, simple, 78–109 × 6–8 ($\bar{X}$ = 86 × 7.5, n = 30) µm. Terminal cell of conidiophores smooth, thin-walled, 18–25 ($\bar{X}$ = 21, n = 30) µm long, bearing a stipe 6–8 ($\bar{X}$ = 7.6, n = 30) µm long, apically swollen; vesicles spatulate to conical, 14–19 × 7–9 ($\bar{X}$ = 17.1 × 8.2, n = 30) µm. Metulae broadly ovoid to broadly ellipsoid, 7–10 × 5–7 ($\bar{X}$ = 7.6 × 6, n = 30) µm, borne on vesicles. Phialides cylindrical to lageniform, 9–15 × 2–3 ($\bar{X}$ = 13.5 × 2.2, n = 30) µm. Vesicles together with metulae and phialides forming spherical to ovoid heads, 37–59 × 28–44 ($\bar{X}$ = 44 × 35, n = 30) µm. Conidia hyaline, smooth, lacrimoid to subclavate, gradually narrowing toward the base, thin-walled, aseptate, 5.5–6.5 × 2–3 ($\bar{X}$ = 6 × 2.5, n = 50) µm. Sexual morph not observed.

*Culture characteristics*. Colonies on PDA grow slowly at 25 °C, reaching 15–19 mm in diameter after 30 days. Mycelium initially greyish-white to cream-white, with colony margins gradually turning pale yellow. The central base darkens, and colonies are compact on the surface. Sporulation not observed in culture.

*Distribution*. China, Jilin Province, Baishan City and Yanbian Korean Autonomous Prefecture.

*Other Material Examined*. China, Jilin Province, Baishan City, Fusong County (42.69° N, 127.40° E; alt. 639 m), on a spider attached to a dead stem, July 2024, Yao Wang (GMB 3154; living culture: GMBC 3154). Yanbian Korean Autonomous Prefecture, Dunhua County (43.68° N, 128.11° E; alt. 647 m), on spiders attached to dead stems, 26 August 2024, Yao Wang (GMB 3155, GMB 3156, GMB 3157; living cultures: GMBC 3155, GMBC 3156, GMBC 3157); 1 September 2024, Yao Wang (GMB 3158, GMB 3159; living cultures: GMBC 3158, GMBC 3159).

*Notes. Gibellula jilinensis* forms a maximally supported sister clade with *G. baishanensis* (IQ-TREE-BS/RAxML-BS/BI-PP = 100%/100%/1), confirming their close relationship while underscoring their distinct species status. Morphologically, *G. jilinensis* is distinguished by synnemata that are restricted to the host’s dorsal surface, unlike in *G. baishanensis* where they also occur on the legs. Furthermore, its synnemata undergo a distinct colour transition during drying, progressing from white to pale yellow, then to deep yellow or brown, and finally to purplish-grey, with spores aggregating dorsally. The species also produces notably smaller conidial heads (37–59 × 28–44 µm) and significantly larger vesicles (14–19 × 7–9 µm) than *G. baishanensis*. These consistent morphological differences, supported by molecular data, validate the recognition of *G. jilinensis* as a new species.

*Gibellula kunmingensis* Y. Wang & H. Chen, sp. nov.

Figure 4.

Mycobank No: 861167

*Etymology*. The species epithet refers to Kunming City in Yunnan Province, China, where the holotype was collected.

*Type*. China, Yunnan Province, Kunming City, Wild Duck Lake Forest Park (25.21° N, 102.85° E; alt. 2110 m), on a spider attached to the underside of a leaf, August 2024, Yao Wang (holotype: GMB 3148; ex-type living culture: GMBC 3148).

*Description*. The host surface is densely covered by a white mycelial mat. Synnemata are white, numerous, and arise directly from the entire host body. The stipes are short, erect, cylindrical, and resemble a narrow head, ca. 5 mm long. Conidiophores numerous, distinctly clustered, white, arising directly from the mycelium covering the host or along the synnemata; erect, thick-walled, septate, with wider spacing between septa toward the apex; simple, with verrucose to globose ornamentation near the apex and conspicuous septal thickening, 52–120 × 14–16 ($\bar{X}$ = 88 × 15, n = 30) µm, abruptly narrowing at the terminal cell to 7–8 ($\bar{X}$ = 7.5, n = 30) µm. Terminal cell of conidiophores smooth, thin-walled, 14–21 ($\bar{X}$ = 18, n = 30) µm long, bearing a stipe 5–7 ($\bar{X}$ = 6, n = 30) µm long, apically swollen. Vesicles spatulate to conical, 13–17 × 5–7 ($\bar{X}$ = 14.5 × 6.5, n = 30) µm, smooth, bearing broadly ovoid to broadly ellipsoid metulae, 13–17 × 5–7 ($\bar{X}$ = 15.5 × 6, n = 30) µm, each supporting cylindrical to lageniform phialides, 12–21 × 7–8 ($\bar{X}$ = 16 × 7, n = 30) µm. Vesicles together with metulae and phialides forming spherical to ovoid heads, 36–49 × 25–35 ($\bar{X}$ = 44 × 31, n = 30) µm, occasionally much reduced in size and complexity. Conidia hyaline, smooth, lacrimoid to subclavate, gradually narrowing toward the base, thin-walled, aseptate, 2–4 × 1.2–2 ($\bar{X}$ = 3.2 × 1.5, n = 50) µm. Sexual morph not observed.

*Culture characteristics*. Colonies on PDA grow slowly at 25 °C, reaching 28–32 mm in diameter after 30 days. Mycelium initially greyish-white to cream-white, with the colony margin gradually turning pale yellow with age. Colonies are loose on the surface but compact at the base. Sporulation not observed in culture.

*Distribution*. Currently known only from Kunming City, Yunnan Province, China.

*Other Material Examined*. China, Yunnan Province, Kunming City (25.35° N, 102.58° E; alt. 1987 m), on spiders attached to dead stems, August 2025, Hui Chen (GMB 3149, GMB 3150, GMB 3151; living cultures: GMBC 3149, GMBC 3150, GMBC 3151).

*Notes*. Morphological comparisons between *G. kunmingensis* and similar species are summarized in Table 2. Although *G. kunmingensis* resembles *G. pulchra* in the production of numerous synnemata, it is distinguished by a combination of shorter synnemata (approximately 5 mm long), spatulate to conical vesicles, and smaller conidial heads (36–49 × 25–35 µm). Furthermore, while other related species listed in Table 2 typically produce paired or multiple synnemata ranging in colour from white to brownish-white, those of *G. kunmingensis* are consistently numerous and milky white. Phylogenetically, *G. kunmingensis* forms an independent clade with moderate support (IQ-TREE-BS/RAxML-BS/BI-PP = 74/94/-). The distinct morphological characteristics, coupled with its phylogenetic isolation, support the recognition of *G. kunmingensis* as a new species.

*Gibellula paralongispora* Y. Wang & H. Chen, sp. nov.

Figure 5.

Mycobank No: 861168

*Etymology.* The specific epithet refers to the morphological resemblance to *Gibellula longispora*.

*Type.* China, Jilin Province, Yanbian Korean Autonomous Prefecture, Dunhua County (43.07° N, 128.01° E; alt. 637 m), on a spider attached to the underside of a leaf, 26 August 2024, Yao Wang (holotype: GMB 3162; ex-type living culture: GMBC 3162).

*Description.* Mycelium covering the host, white to cream, floccose, becoming light greyish brown to violaceous-brown upon drying. Synnemata multiple, cylindrical, arising from the abdomen of the host spider, cream to yellowish white. Conidiophores 111–188 × 8–10 ($\bar{X}$ = 168 × 9, n = 30) μm, densely arranged, arising secondarily from hyphae loosely attached to the synnematal surface; verrucose, multiseptate, abruptly narrowing at the apex and forming a globose vesicle, 8–10.5 × 5–8 ($\bar{X}$ = 9.5 × 6, n = 30) μm. Conidial heads spherical, composed of vesicles, metulae, and phialides, 28.5–40 × 25–36.5 ($\bar{X}$ = 37 × 32, n = 30) μm. Metulae broadly obovate to oval, 6–11 × 4–7 ($\bar{X}$ = 8 × 5.5, n = 30) μm, borne on vesicles, each bearing several clavate phialides, 9–11 × 2–5 ($\bar{X}$ = 10 × 4, n = 30) μm. Conidia narrowly fusiform, 4.5–6 × 1.5–2 ($\bar{X}$ = 5 × 1.8, n = 50) μm. Neither teleomorph nor granulomanus-like synanamorph observed.

*Culture characteristics*. Colonies on PDA grow slowly at 25 °C, reaching 18–21 mm in diameter after 30 days. Mycelium initially greyish-white to cream-white, with margins gradually turning pale yellow to brown with age. Colonies are compact both superficially and at the base. Sporulation not observed in culture.

*Distribution*. Currently known from Baishan City and Yanbian Korean Autonomous Prefecture in Jilin Province, and Tieling City in Liaoning Province, China.

*Other Material Examined.* China, Jilin Province, Baishan City, Fusong County (42°37′ N, 127°59′ E; alt. 639 m), on a spider attached to the underside of a leaf, 29 July 2025, collected by Kun Zhang (GMB 3161; living culture: GMBC 3161); Yanbian Korean Autonomous Prefecture, Dunhua County (43.10° N, 128.05° E; alt. 630 m), on spiders attached to the underside of leaves, 26 August 2025, Kun Zhang (GMB 3163, GMB 3164; living culture: GMBC 3163, GMBC 3164); Liaoning Province, Tieling City, Xifeng County (42°54′ N, 124°41′ E; alt. 346 m), on a spider attached to the underside of a leaf, 25 September 2025, collected by Kun Zhang (GMB 3165; living culture: not available).

*Notes. Gibellula paralongispora* forms a sister clade to *G. longispora* in the phylogeny, with strong support (IQ-TREE-BS/RAxML-BS/BI-PP = 97%/96%/0.99). Morphologically, both species share similar conidiophore structures and globose conidial heads. However, *G. paralongispora* differs in its narrower conidia (4.5–6 × 1.5–2 μm vs. 5–7 × 1–2 μm in *G. longispora*) and longer conidiophores (111–188 μm vs. 60–153.5 μm). These consistent morphological distinctions, together with molecular phylogenetic evidence, support the recognition of *G. paralongispora* as a distinct species.

*Gibellula yunnanensis* Y. Wang & H. Chen, sp. nov.

Figure 6.

Mycobank No: 861190

*Etymology*. The specific epithet refers to Yunnan Province, China, where the species was collected.

*Type*. China, Yunnan Province, Kunming City, Wild Duck Lake Forest Park (25.68*°* N, 102.47*°* E, alt. 2100 m), on a spider attached to the underside of a leaf, 26 August 2024, Yao Wang (holotype: GMB 3142; ex-type living culture: not available).

*Description*. Mycelium covering the host, white to creamy yellow, becoming light greyish-brown to violaceous-brown when dried. Synnemata multiple, cylindrical, arising from the abdomen of the host spider, cream to yellowish white. Conidiophores 27–58 × 3–8 ($\bar{X}$ = 33 × 6, n = 30) μm, crowded, arising from hyphae loosely attached to the surface of the synnema, verrucose, multiseptate, suddenly narrowing to a tip, and forming a globose vesicle, 6–9 × 5.5–8 ($\bar{X}$ = 7.5 × 6, n = 30) μm. Conidial heads composed of vesicle, metulae, and phialides, 22.5–31 × 36–46.5 ($\bar{X}$ = 25 × 42, n = 30) μm. Metulae broadly obovate to oval, 6.4–9.7 × 5–7 ($\bar{X}$ = 8 × 6, n = 30) μm, borne on vesicles. Phialides clavate, 6–8 × 2–3 ($\bar{X}$ = 7 × 2.2, n = 30) μm, several per metula. Conidia narrowly fusiform, 6–9 × 2.2–3.1 ($\bar{X}$ = 7.6 × 2.8, n = 50) μm. Teleomorph and granulomanus-like synanamorphs not observed.

*Distribution*. Currently known from Kunming City and Puer City in Yunnan Province, China.

*Other Material Examined*. China, Yunnan Province, Puer City (22.58° N, 99.97° E, alt. 1287 m), on a spider attached to the underside of a leaf, August 2025, Hui Chen (GMB 3143; living culture: not available).

*Notes*. In the phylogeny, *G. yunnanensis* clusters with *G. attenboroughii* and *G. flava*, forming a highly supported sister clade with *G. attenboroughii* (IQ-TREE-BS/RAxML-BS/BI-PP = 99%/90%/0.92). Morphologically, *G. yunnanensis* resembles *G. attenboroughii* and *G. flava* in producing multiple synnemata and aspergillate, distinctly roughened conidiophores. However, *G. yunnanensis* differs from *G. attenboroughii* in several stable morphological features. The conidia of *G. yunnanensis* are distinctly longer and narrower, whereas those of *G. attenboroughii* are ellipsoidal to fusoid (6–9 × 2.2–3.1 μm vs. 4–6 × 1.5–2 μm). In addition, the conidial heads of *G. yunnanensis* are densely arranged, while those of *G. attenboroughii* are relatively loose. These consistent morphological distinctions, combined with its stable phylogenetic position, support the recognition of *G. yunnanensis* as an independent species.

## 4. Discussion

In China, research on spider-pathogenic fungi has a relatively long history, yet the diversity of *Gibellula* has only recently gained fuller appreciation. By the 1980s, only a single species—initially reported as *G. pulchra* [51]—was known. This record was later revised to *G. leiopus*, a species characterized by extremely short conidiophores that confer a compact appearance [20]. Subsequent studies in the 1990s described several new taxa from Taiwan and Anhui Province, and over the past decade, systematic work has significantly expanded the known diversity [40,45,50]. To date, a total of 14 species and/or varieties of *Gibellula* have been reported from China, including *G. clavispora*, *G. clavulifera*, *G. clavulifera* var. *major*, *G. curvispora*, *G. dabieshanensis*, *G. dimorpha*, *G. flava*, *G. leiopus*, *G. longispora*, *G. penicillioides*, *G. pulchra*, *G. shennongjiaensis*, *G. unica*, and *G. liaoningensis* [14,17,19,45,50,52]. Among these, *G. pulchra* and *G. leiopus* are commonly encountered in southern China. The discovery of the five new species in this study, particularly those from Jilin and Liaoning Provinces, therefore significantly contributes new distribution records for *Gibellula* in Northeast China and underscores the ongoing potential for taxonomic discovery even in previously studied geographic contexts.

Host specificity offers critical insights for evaluating both the virulence of pathogens and their potential application as biological control agents [40]. Concurrently, increasing attention is being directed towards the secondary metabolites produced by *Gibellula* species. For instance, the novel antimicrobial compound EPF083CE was isolated from *G. pulchra* EPF083 [53]; pigmentosins A and B were obtained from the spider-associated fungus *G. pigmentosinum* [54]; and gibellamines A and B were characterized from *G. gamsii* [43]. Notably, pigmentosin B and gibellamines appear to be unique to *G. pigmentosinum* and *G. gamsii*, respectively, suggesting their potential utility as chemotaxonomic markers [40].

Members of *Gibellula* are notoriously fastidious and often challenging to establish in pure culture. In this study, however, we achieved successful isolation of the majority of the newly described taxa, with four out of the five species grown on potato dextrose agar (PDA) from conidial sources, despite their generally slow growth rates. This cultivation success represents a significant step forward, paving the way for utilizing these isolates in the discovery of novel bioactive metabolites and for identifying chemical markers valuable in taxonomic studies.

## Figures and Tables

**Figure 2:**
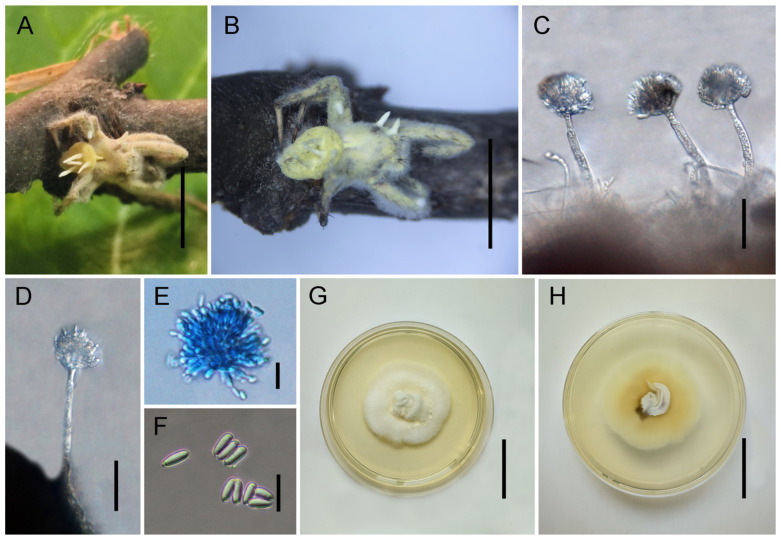
Morphology of *Gibellula baishanensis.* (**A**,**B**) Fungus on a spider. (**C**–**E**) Conidiophores showing conidial heads. (**F**) Conidia. (**G**,**H**) Colonies on PDA (front and reverse). Scale bars: 10 mm (**A**,**B**); 100 µm (**C**,**D**); 30 µm (**E**); 10 µm (**F**); 20 mm (**G**,**H**).

**Figure 3:**
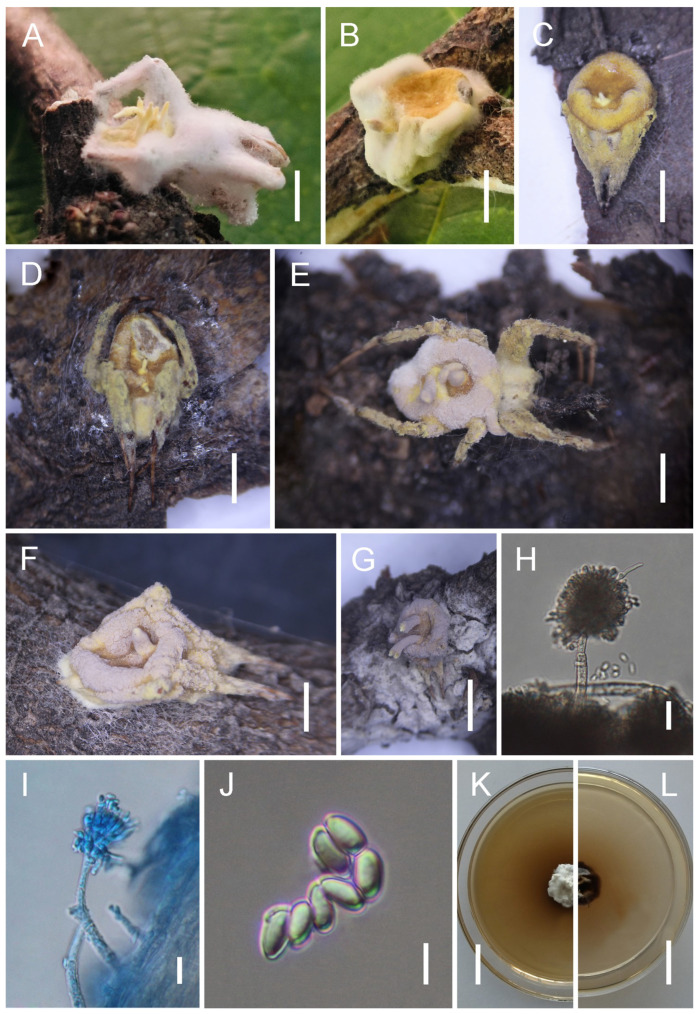
Morphology of *Gibellula jilinensis.* (**A**–**G**) Fungus on a spider. (**H**,**I**) Conidiophores showing conidial heads. (**J**) Conidia. (**K**,**L**) Colony on PDA(front and reverse). Scale bars: 10 mm (**A**–**G**); 40 µm (**H**,**I**); 5 µm (**J**); 15 mm (**K**,**L**).

**Figure 4:**
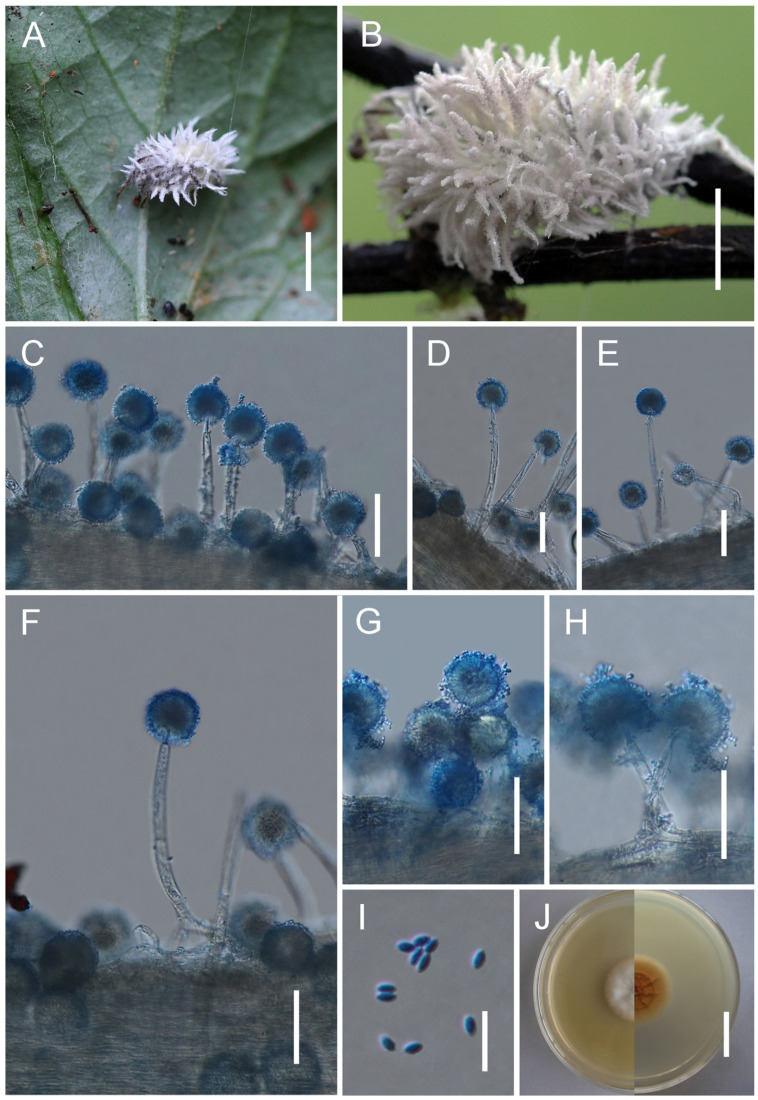
Morphology of *Gibellula kunmingensis.* (**A**,**B**) Fungus on a spider. (**C**–**H**) Conidiophores showing conidial heads. (**I**) Conidia. (**J**) Colonies on PDA (front and reverse). Scale bars: 10 mm (**A**,**B**); 50 µm (**C**–**H**); 10 µm (**I**); 20 mm (**J**).

**Figure 5:**
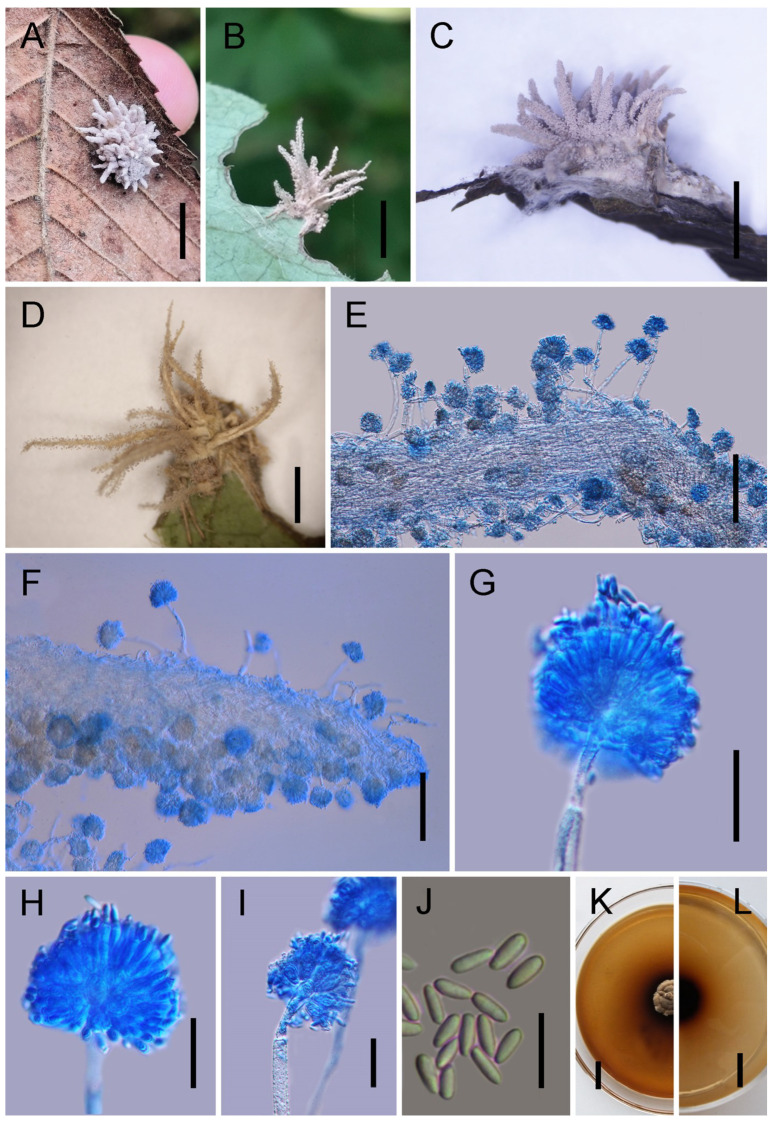
Morphology of *Gibellula paralongispora.* (**A**–**D**) Fungus on a spider. (**E**–**I**) Conidiophores showing conidial heads. (**J**) Conidia. (**K**,**L**) Colony on PDA(front and reverse). Scale bars: 10 mm (**A**,**B**); 5 mm (**C**,**D**); 100 µm (**E**,**F**); 20 µm (**G**–**I**); 10 µm (**J**); 15 mm (**K**,**L**).

**Figure 6:**
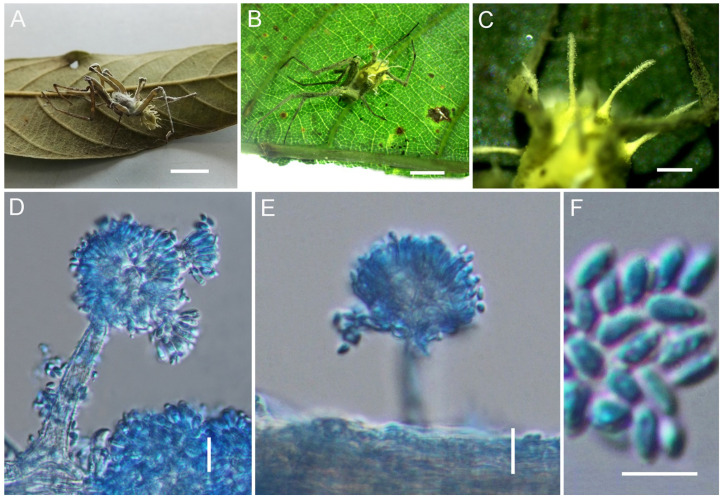
Morphology of *Gibellula yunnanensis*. (**A**–**C**) Fungus on a spider. (**D**,**E**) Conidiophores showing conidial heads. (**F**) Conidia. Scale bars: 10 mm (**A**–**C**); 10 µm (**D**–**F**).

**Table 1:** Relevant species information and GenBank accession numbers for phylogenetic research in this study.

Species	Voucher/Information	GenBank Accession Number						References
		nr*SSU*	ITS	nr*LSU*	*tef-1α*	*rpb1*	*rpb2*	
*Blackwellomyces kaihuaensis*	HMAS 285455^T^	OQ981975	OQ981961	OQ981968	OQ980401	OQ980409	OQ980408	[35]
*Blackwellomyces lateris*	MFLU 18-0663^T^	MK086057	MK086059	MK086061	MK069471	MK084615	MK079354	[36]
*Gibellula agrofloretalis*	A30	PP958494	N/A	N/A	PP965288	N/A	N/A	[37]
*Gibellula agrofloretalis*	C11	PP958496	N/A	N/A	PP965293	N/A	N/A	[37]
*Gibellula agrofloretalis*	D7	PP958504	N/A	PP958435	PP965304	N/A	N/A	[37]
*Gibellula attenboroughii*	IMI 507230^T^	PQ036924	N/A	PQ036929	PQ046101	N/A	N/A	[38]
*Gibellula aurea*	LBMCF0003	OK329880	N/A	N/A	OK392618	N/A	OL117022	[39]
*Gibellula aurea*	LBMCF0006	N/A	N/A	OK329875	OK392624	N/A	OK315662	[39]
*Gibellula aurea*	LBMCF0007	N/A	OK329885	OK329876	OK392622	N/A	OK315663	[39]
***Gibellula baishanensis***	**GMBC 3152^T^**	**PX425053**	**PX425062**	**PX425069**	**PX434402**	**PX434411**	**PX434420**	**This study**
***Gibellula baishanensis***	**GMBC 3153**	**PX425054**	**PX425063**	**PX425070**	**PX434403**	**PX434412**	**PX434421**	**This study**
*Gibellula brevistipitata*	BCC 45580^T^	N/A	OK040729	OK040706	OK040697	OK040715	N/A	[7]
*Gibellula cebrennini*	BCC 53605^T^	N/A	MT477069	MT477062	MT503328	MT503321	MT503336	[40]
*Gibellula cebrennini*	BCC 39705	N/A	MH532874	MH394673	MH521895	MH521822	MH521859	[40]
*Gibellula clavulifera* var. *alba*	ARSEF 1915^T^	DQ522562	JN049837	DQ518777	DQ522360	DQ522408	DQ522467	[41,42]
*Gibellula dimorpha*	BCC 47518	N/A	MH532884	MH394679	MH521892	MH521819	MH521863	[7]
*Gibellula flava*	GNJ20200814-46	MW969660	N/A	MW969673	MW961413	MW980146	N/A	[18]
*Gibellula flava*	WFS20190625-25^T^	MW036749	N/A	MW084343	MW091325	MW384883	N/A	[18]
*Gibellula fusiformispora*	BCC 56802^T^	N/A	MT477070	MT477063	MT503329	MT503322	MT503337	[40]
*Gibellula fusiformispora*	BCC 45076	N/A	MH532882	N/A	N/A	MH521823	MH521860	[40]
*Gibellula gamsii*	BCC 27968^T^	N/A	MH152529	MH152539	MH152560	MH152547	N/A	[43]
*Gibellula gamsii*	BCC 29228	N/A	MH152533	MH152543	MH152564	MH152551	MH152558	[43]
*Gibellula gamsii*	EPF034	N/A	JX192720	JX192753	JX192817	N/A	N/A	[43]
***Gibellula jilinensis***	**GMBC 3154**	**PX425050**	**PX425059**	**PX425066**	**PX434399**	**PX434408**	**PX434417**	**This study**
***Gibellula jilinensis***	**GMBC 3157**	**PX425051**	**PX425060**	**PX425067**	**PX434400**	**PX434409**	**PX434418**	**This study**
***Gibellula jilinensis***	**GMBC 3160^T^**	**PX425052**	**PX425061**	**PX425068**	**PX434401**	**PX434410**	**PX434419**	**This study**
***Gibellula*** ** ** ***kunmingensis***	**GMBC 3148^T^**	**PX425057**	**PX425064**	**PX425073**	**PX434406**	**PX434415**	**PX434424**	**This study**
***Gibellula*** ** ** ***kunmingensis***	**GMBC 3149**	**PX425058**	**PX425065**	**PX425074**	**PX434407**	**PX434416**	**PX434425**	**This study**
*Gibellula leiopus*	BCC 16025	N/A	N/A	MF416548	MF416492	MF416649	N/A	[44]
*Gibellula leiopus*	BCC 49250	N/A	OK070780	OK070781	OK070782	OK070783	OK070784	[7]
*Gibellula liaoningensis*	HKAS 145357	PQ817100	PQ817098	PQ817102	PQ815114	PQ815116	PQ815118	[45]
*Gibellula liaoningensis*	HKAS 145358^T^	PQ817099	PQ817097	PQ817101	PQ815113	PQ815115	PQ815117	[45]
*Gibellula longicaudata*	BCC 40861	N/A	OK040730	OK040707	OK040698	OK040716	OK040724	[7]
*Gibellula longispora*	NHJ 12014	EU369098	N/A	N/A	EU369017	EU369055	EU369075	[46]
*Gibellula longispora*	GNJ20200813-16	N/A	N/A	N/A	MW961414	MW980145	N/A	[18]
*Gibellula longispora*	GNJ20210710-02	OL854201	N/A	OL854212	OL981628	N/A	OL981635	[18]
*Gibellula mainsii*	LBMCF2022.96	OQ585789	OQ589484	N/A	OQ658392	N/A	N/A	[47]
*Gibellula mirabilis*	LBMCF2021.70	OQ585786	OQ589481	OQ585976	OQ658389	N/A	N/A	[47]
*Gibellula mirabilis*	LBMCF2021.80	OQ585787	OQ589482	OQ585977	OQ658390	N/A	N/A	[47]
*Gibellula mirabilis*	LBMCF2022.107	OQ585792	N/A	OQ585979	OQ658395	N/A	N/A	[47]
*Gibellula nigelii*	NHJ 10808^T^	EU369099	N/A	EU369035	EU369018	EU369056	EU369076	[46]
***Gibellula paralongispora***	**GMBC 3162^T^**	**PX425055**	**N/A**	**PX425071**	**PX434404**	**PX434413**	**PX434422**	**This study**
***Gibellula paralongispora***	**GMBC 3163**	**PX425056**	**N/A**	**PX425072**	**PX434405**	**PX434414**	**PX434423**	**This study**
*Gibellula parvula*	BCC 48888	N/A	OK040731	OK040708	OK040699	OK040717	OK040725	[7]
*Gibellula parvula*	BCC 49748^T^	N/A	OK040732	OK040709	OK040700	OK040718	OK040726	[7]
*Gibellula penicillioides*	GNJ20200814-11	MW969650	MW969669	MW969661	MW961415	MZ215998	N/A	[18]
*Gibellula penicillioides*	GNJ20200814-14^T^	MW969651	MW969670	MW969662	MW961416	MZ215999	N/A	[18]
*Gibellula penicillioides*	GNJ20200814-17	MW969652	MW969671	MW969663	MW961417	N/A	N/A	[18]
*Gibellula pigmentosinum*	BCC 38246	N/A	MH532872	MH394672	MH521893	MH521800	MH521855	[40]
*Gibellula pigmentosinum*	BCC 41203^T^	N/A	MT477071	MT477064	MT503330	MT503323	N/A	[40]
*Gibellula pilosa*	BCC 57817^T^	N/A	OK040733	OK040710	OK040701	OK040719	N/A	[7]
*Gibellula pulchra*	BCC 47555	N/A	MH532885	N/A	MH521897	MH521804	N/A	[7]
*Gibellula pulchra*	LBMCF2020.02	OQ585783	N/A	OQ585973	OQ658386	N/A	N/A	[47]
*Gibellula pulchra*	LBMCF2020.03	OQ585784	N/A	OQ585974	OQ658387	N/A	N/A	[47]
*Gibellula pulchra*	LBMCF2020.07	OQ585785	N/A	OQ585975	OQ658388	N/A	N/A	[47]
*Gibellula pulchra*	LBMCF2022.GA	OQ585780	N/A	OQ585970	OQ658383	N/A	N/A	[47]
*Gibellula pulchra*	LBMCF2022.GB	OQ585781	OQ589487	OQ585971	OQ658384	N/A	N/A	[47]
*Gibellula queenslandica*	BRIP 72767a^T^	N/A	OR452099	OR452103	OR459912	N/A	OR459907	[48]
*Gibellula scorpioides*	BCC 47976^T^	N/A	MT477078	MT477066	MT503335	MT503325	MT503339	[40]
*Gibellula solita*	BCC 45574^T^	N/A	OK040736	OK040712	OK040703	OK040721	N/A	[7]
*Gibellula* sp.	NHJ 5401	EU369102	N/A	N/A	N/A	EU369059	EU369079	[46]
*Gibellula* sp.	NHJ 10788	EU369101	N/A	EU369036	EU369019	EU369058	EU369078	[46]
*Gibellula trimorpha*	BCC 36526^T^	N/A	OK040737	N/A	OK040704	OK040722	OK040728	[7]
*Gibellula trimorpha*	BCC 36538	N/A	MH532867	MH394668	MH521890	MH521817	MH521861	[7]
*Gibellula unica*	BCC 45112	N/A	OK040738	N/A	OK040705	OK040723	N/A	[7]
*Gibellula unica*	BCC 46590	N/A	MH532883	MH394678	N/A	MH521803	MH521866	[7]
***Gibellula yunnanensis***	**GMB 3142^T^**	**PX354537**	**PX354531**	**PX354543**	**PX370035**	**PX371911**	**PX371921**	**This study**
***Gibellula yunnanensis***	**GMB 3143**	**PX354538**	**PX354532**	**PX354544**	**PX370036**	**PX371912**	**PX371922**	**This study**
*Hevansia arachnophila*	NHJ 2633	N/A	MH532900	GQ249978	MH521917	MH521843	MH521884	[43]
*Hevansia minula*	BCC 47519^T^	N/A	MZ684087	MZ684002	MZ707811	MZ707826	MZ707833	[9]
*Hevansia minula*	BCC 47520	N/A	MZ684088	MZ684003	MZ707812	MZ707827	MZ707834	[9]
*Hevansia nelumboides*	TNS 16306	MF416585	N/A	N/A	MF416475	N/A	MF416438	[44]
*Hevansia novoguineensis*	BCC 42675	N/A	MZ684089	MZ684004	MZ707814	N/A	MZ707835	[9]
*Hevansia novoguineensis*	CBS 610.80^T^	N/A	MH532831	MH394646	MH521885	N/A	MH521844	[49]
*Jenniferia cinerea*	NHJ 03510^T^	N/A	N/A	N/A	EU369009	EU369048	EU369070	[46]
*Jenniferia cinerea*	BCC 2191	GQ249956	GQ250000	GQ249971	GQ250029	N/A	N/A	[43]
*Jenniferia griseocinerea*	BCC 42062^T^	N/A	MZ684091	MZ684006	MZ707815	MZ707828	MZ707837	[9]
*Jenniferia griseocinerea*	BCC 42063	N/A	MZ684092	MZ684007	MZ707816	MZ707829	MZ707838	[9]
*Jenniferia thomisidarum*	BCC 37881^T^	N/A	MZ684099	MZ684010	MZ707823	MZ707830	MZ707843	[9]
*Jenniferia thomisidarum*	BCC 37882	N/A	MZ684100	MZ684011	MZ707824	MZ707831	MZ707844	[9]

**Boldface**: data generated in this study; **^T^**: ex-type material. Institutional acronyms: **ARSEF**: Agricultural Research Service Collection of Entomopathogenic Fungal Cultures (culture collection); **BCC**: BIOTEC Culture Collection (culture collection); **CBS**: Westerdijk Fungal Biodiversity Institute (culture collection); **GMB**: Herbarium of Guizhou Medical University (herbarium); **GMBC**: Guizhou Medical University Culture Collection (culture collection); **HKAS**: Herbarium of Cryptogams, Kunming Institute of Botany, Chinese Academy of Sciences (herbarium); **IMI**: CABI Bioscience UK Centre (includes both cultures and herbarium specimens); **NHJ**: National Herbarium of Japan (herbarium); **TNS**: National Museum of Nature and Science (herbarium); **MFLU**: Mae Fah Luang University (herbarium).

**Table 2:** Comparison of the morphological characters of *G. baishangensis*, *G. jilinensis*, *G. kunmingensis*, *G. paralongispora* and related species.

Species	Conidiophore (μm)	Metulae (μm)	Phialide (μm)	Conidia (μm)	References
*Gibellula attenboroughii*	Verrucose, 80–120 × 5–8	Borne on vesicle, 10–12 × 6–8	Cylindrical to narrowly clavate, 7.5–9.5 × 2.5–3.5	Hyaline, smooth, 4–6 × 1.5–2	[38]
***G. baishangensis***	**Verrucose to globose, 69–89 × 8–11**	**Broadly ovoid to broadly ellipsoid, 5–11 × 6–7**	**Spherical to ovoid, 10–14 × 2–3**	**Hyaline, smooth, 5.5–7 × 2–4**	**This study**
*G. flava*	Verrucose, 33.5–123.5 × 4–9.5	Obovoid to broadly obovoid, 5.5–7 × 3.5–5.5	Narrowly obovate to clavate, 5.5–7 × 1.5–2.5	Fusiform, 3–4 × 1–2	[50]
***G. jilinensis***	**Verrucose, 78–109 × 6–8**	**Broadly ovoid to broadly ellipsoid, 7–10 × 5–7**	**Cylindrical to lageniform, 9–15 × 2–3**	**Hyaline, smooth, 5.5–6.5 × 2–3**	**This study**
***G. kunmingensis***	**Verrucose to globose, 52–120 × 14–16**	**Broadly ovoid to broadly ellipsoid, 13–17 × 5–7**	**Cylindrical to lageniform, borne on vesicles, 12–21 × 7–8**	**Hyaline, smooth, 2–4 × 1.2–2**	**This study**
*G. longispora*	Multiseptate, minutely roughened, 159.5–290.5 × 8.5–11	Broadly obovoid, 7.5–9.5 × 6–6.5	Narrowly clavate to cylindrical, 9.5–11 × 3–3.5	Bacilliform to cylindrical, 5.5–8 × 1–1.5	[8]
***G. paralongispora***	**Verrucose, 111–188 × 8–10**	**Broadly obovate to oval, 6–11 × 4–7**	**Clavate, 9–11 × 2–5**	**Narrowly fusiform, 4.5–6 × 1.5–2**	**This study**
*G. penicillioides*	Penicillate, smooth, mostly biverticillate or terverticillate, 52.5–92 × 4.5–6	Obovoid to cylindrical, 13–17.5 × 3.5–5	Broadly cylindrical, 12.5–15.5 × 3–4	7.5–9 × 2.5–3.5	[18]
*G. pigmentosinum*	Smooth to verrucose, 97.5–170 × 7–10	Broadly obovoid, 6–8 × 4–6	Obovoid to clavate, 5.5–8 × 2–3	Obovoid with an acute Apex 3.5–5 × 1–2	[40]
*G. pilosa*	Minutely roughened, 151–265 × 9–11	Broadly obovoid, 9.5–11 × 7–8	Narrowly clavate to cylindrical, 7–9 × 2.5–3	Narrowly almond shaped, 3–4 × 1.5–2	[8]
*G. pulchra*	Verrucose, 155–170 × 7.5–10	Cylindrical, 6.2–7.5 × 5–6	Clavate, 7.5–8 × 1.5–2.5	Fusiform to fusiform ellipsoid, 3–5 × 1.5–2.5	[17]
*G. solita*	Verrucose, 82–146 × 7.5–9.5	Broadly obovoid, 7–7.5 × 5–6	Narrowly clavate to cylindrical, 6–7 × 2–2.5	Occasionally globose, 2–2.5 × 1–1.5	[8]
***G. yunnanensis***	**Verrucose, 27–58 × 3–8**	**Broadly obovate to oval, 6.4–9.7 × 5–7**	**Borne on vesicle, 6.4–9.7 × 5–7**	**Narrowly fusiform, 6–9 × 2.2–3.1**	**This study**

Note: Bold labels indicate the morphological data of the new species described in this study.

## Data Availability

The DNA sequence data obtained in this study have been deposited in GenBank. The accession numbers can be found in the article (Table 1).

## References

[1] Maharachchikumbura S.S., Hyde K.D., Jones E.B., McKenzie E.H., Huang S., Abdel-Wahab M.A., Daranagama D.A., Dayarathne M.C., D’souza M.J., Goonasekara I.D. (2015). Towards a natural classification and backbone tree for *Sordariomycetes*. Fungal Divers..

[2] Wijayawardene N.N., Hyde K.D., Lumbsch H.T., Liu J., Maharachchikumbura S.S., Ekanayaka A.H., Tian Q., Phookamsak R. (2018). Outline of *Ascomycota*: 2017. Fungal Divers..

[3] Xiao Y.P., Wen T.C., Hongsanan S., Jeewon R., Luangsa-Ard J.J., Brooks S., Wanasinghe D.N., Long F.Y., Hyde K.D. (2018). Multigene phylogenetics of *Polycephalomyces* (*Ophiocordycipitaceae*, *Hypocreales*), with two new species from Thailand. Sci. Rep..

[4] Xiao Y.P., Wang Y.B., Hyde K.D., Eleni G., Sun J.Z., Yang Y., Meng J., Yu H., Wen T.C. (2023). *Polycephalomycetaceae*, a new family of clavicipitoid fungi segregates from *Ophiocordycipitaceae*. Fungal Divers..

[5] Wei D.P., Wanasinghe D.N., Xu J.C., To-Anun C., Mortimer P.E., Hyde K.D., Elgorban A.M., Madawala S., Suwannarach N., Karunarathna S.C. (2021). Three novel entomopathogenic fungi from China and Thailand. Front. Microbiol..

[6] Wei D.P., Gentekaki E., Wanasinghe D., Tang S., Hyde K.D. (2023). Diversity, molecular dating and ancestral character state reconstruction of entomopathogenic fungi in *Hypocreales*. Mycosphere.

[7] Peng X.C., Wen T.C., Wei D.P., Liao Y.H., Wang Y., Zhang X., Wang G.Y., Zhou Y., Tangtrakulwanich K., Liang J.D. (2024). Two new species and one new combination of *Ophiocordyceps* (*Hypocreales*, *Ophiocordycipitaceae*) in Guizhou. MycoKeys.

[8] Kuephadungphan W., Petcharad B., Tasanathai K., Thanakitpipattana D., Kobmoo N., Khonsanit A., Samson R.A., Luangsa-Ard J.J. (2022). Multilocus phylogeny unmasks hidden species within the specialised spider-parasitic fungus, *Gibellula* (*Hypocreales*, *Cordycipitaceae*) in Thailand. Stud. Mycol..

[9] Shrestha B., Kubátová A., Tanaka E., Oh J., Yoon D.H., Sung J.M., Sung G.H. (2019). Spider-pathogenic fungi within *Hypocreales* (*Ascomycota*): Their current nomenclature, diversity, and distribution. Mycol. Prog..

[10] Mongkolsamrit S., Noisripoom W., Tasanathai K., Kobmoo N., Thanakitpipattana D., Khonsanit A., Petcharad B., Himaman W. (2022). Comprehensive treatise of *Hevansia* and three new genera *Jenniferia*, *Parahevansia* and *Polystromomyces* on spiders in *Cordycipitaceae* from Thailand. MycoKeys.

[11] Joseph R.A., Masoudi A., Valdiviezo M.J., Keyhani N.O. (2024). Discovery of *Gibellula floridensis* from infected spiders and analysis of the surrounding fungal entomopathogen community. J. Fungi.

[12] Khonsanit A., Thanakitpipattana D., Mongkolsamrit S., Kobmoo N., Phosrithong N., Samson R.A., Crous P.W., Luangsa-Ard J.J. (2024). A phylogenetic assessment of *Akanthomyces* sensu lato in *Cordycipitaceae* (*Hypocreales*, *Sordariomycetes*): Introduction of new genera, and the resurrection of *Lecanicillium*. Fungal Syst. Evol..

[13] Samson R.A., Evans H.C. (1973). Notes on entomogenous fungi from Ghana: I. The genera *Gibellula* and *Pseudogibellula*. Acta Bot. Neerl..

[14] Huang B., Ding D.G., Fan M.Z., Li Z.Z. (1998). A new entomopathogenic fungus on spiders. Mycosystema.

[15] Han Y.F., Chen W.H., Zou X., Liang Z.Q. (2013). *Gibellula curcispora*, a new species of *Gibellula*. Mycosystema.

[16] Chen W.H., Han Y.F., Liang Z.Q., Wang Y.R., Zou X. (2014). Classification of *Gibellula* spp. by DELTA system. Microbiol. China.

[17] Chen W.H., Han Y.F., Liang Z.Q., Zou X. (2016). Morphological traits, DELTA system, and molecular analysis for *Gibellula clavispora* sp. nov. from China. Mycotaxon.

[18] Chen M., Wang T., Lin Y., Huang B. (2022). Morphological and molecular analyses reveal two new species of *Gibellula* (*Cordycipitaceae*, *Hypocreales*) from China. MycoKeys.

[19] Zou X., Chen W.H., Han Y.F., Liang Z.Q. (2016). A new species of the genus *Gibellula*. Mycosystema.

[20] Wang T., Chang X.Y., Huang B., Li Z.Z., Chen M.J. (2024). Species diversity of spider-pathogenic fungi in China. Mycosystema.

[21] Sun T., Zou W.Q., Dong Q.Y., Huang O., Tang D.X., Yu H. (2022). Morphology, phylogeny, mitogenomics and metagenomics reveal a new entomopathogenic fungus *Ophiocordyceps nujiangensis* (*Hypocreales*, *Ophiocordycipitaceae*) from Southwestern China. MycoKeys.

[22] Wang Y.B., Wang Y., Fan Q., Duan D.E., Zhang G.D., Dai R.Q., Dai Y.D., Zeng W.B., Chen Z.H., Li D.D. (2020). Multigene phylogeny of the family *Cordycipitaceae* (*Hypocreales*): New taxa and the new systematic position of the Chinese cordycipitoid fungus *Paecilomyces hepiali*. Fungal Divers..

[23] White T.J., Bruns T., Lee S., Taylor J.W. (1990). Amplification and direct sequencing of fungal ribosomal RNA genes for phylogenetics. PCR Protocols: A Guide to Methods and Applications.

[24] Rehner S.A., Samuels G.J. (1994). Taxonomy and phylogeny of *Gliocladium* analysed from nuclear large subunit ribosomal DNA sequences. Mycol. Res..

[25] Bischoff J.F., Rehner S.A., Humber R.A. (2006). *Metarhizium frigidum* sp. nov.: A cryptic species of *M. anisopliae* and a member of the *M. flavoviride* complex. Mycologia.

[26] Sung G.H., Hywel-Jones N.L., Sung J.M., Luangsa-ard J.J., Shrestha B., Spatafora J.W. (2007). Phylogenetic classification of *Cordyceps* and the clavicipitaceous fungi. Stud. Mycol..

[27] Liu Y.J., Whelen S., Hall B.D. (1999). Phylogenetic relationships among ascomycetes: Evidence from an RNA polymerase II subunit. Mol. Biol. Evol..

[28] Castlebury L.A., Rossman A.Y., Sung G.H., Hyten A.S., Spatafora J.W. (2004). Multigene phylogeny reveals new lineage for *Stachybotrys chartarum*, the indoor air fungus. Mycol. Res..

[29] Wang Y.B., Yu H., Dai Y.D., Chen Z.H., Zeng W.B., Yuan F., Liang Z.Q. (2015). *Polycephalomyces yunnanensis* (*Hypocreales*), a new species of *Polycephalomyces* parasitizing *Ophiocordyceps nutans* and stink bugs (hemipteran adults). Phytotaxa.

[30] Tamura K., Stecher G., Peterson D., Filipski A., Nei M., Kumar S. (2013). MEGA6: Molecular evolutionary genetics analysis version 6.0. Mol. Biol. Evol..

[31] Stamatakis A., Hoover P., Rougemont J. (2008). A rapid bootstrap algorithm for the RAxML Web servers. Syst. Biol..

[32] Minh B.Q., Schmidt H.A., Chernomor O., Schrempf D., Woodhams M.D., von Haeseler A., Lanfear R. (2020). IQ-TREE 2: New models and efficient methods for phylogenetic inference in the genomic era. Mol. Biol. Evol..

[33] Darriba D., Taboada G.L., Doallo R., Posada D. (2012). jModelTest 2: More models, new heuristics and parallel computing. Nat. Methods.

[34] Ronquist F., Teslenko M., van der Mark P., Ayres D.L., Darling A., Höhna S., Larget B., Liu L., Suchard M.A., Huelsenbeck J.P. (2012). MrBayes 3.2: Efficient Bayesian phylogenetic inference and model choice across a large model space. Syst. Biol..

[35] Li Y., Zhao X.C., Wu L.X., Wang Y., Xu A., Lin W.F. (2023). *Blackwellomyces kaihuaensis* and *Metarhizium putuoense* (*Hypocreales*), two new entomogenous fungi from subtropical forests in Zhejiang Province, Eastern China. Forests.

[36] Hyde K.D., Tennakoon D.S., Jeewon R.D., Bhat J., Maharachchikumbura S.S.N., Rossi W., Leonardi M., Lee H.B., Mun H.Y., Houbraken J. (2019). Fungal diversity notes 1036–1150: Taxonomic and phylogenetic contributions on genera and species of fungal taxa. Fungal Divers..

[37] Alves J.E.R., Santos A.C.S., Belo Pedroso S.K., Ribeiro Melo R.F., Tiago P.V. (2025). Untangling a web of spider fungi: *Gibellula agroflorestalis* (*Hypocreales*, *Ascomycota*), a new species of spider parasite from Brazil. J. Invertebr. Pathol..

[38] Evans H.C., Fogg T., Buddie A.G., Yeap Y.T., Araújo J.P.M. (2025). The araneopathogenic genus *Gibellula* (*Cordycipitaceae*: *Hypocreales*) in the British Isles, including a new zombie species on orb-weaving cave spiders (*Metainae*: *Tetragnathidae*). Fungal Syst. Evol..

[39] Mendes-Pereira T., de Araújo J.P.M., Mendes F., Fonseca E. (2022). *Gibellula aurea* sp. nov. (*Ascomycota*, *Cordycipitaceae*): A new golden spider-devouring fungus from a Brazilian Atlantic Rainforest. Phytotaxa.

[40] Kuephadungphan W., Tasanathai K., Petcharad B., Khonsanit A., Stadler M., Luangsa-ard J.J. (2020). Phylogeny- and morphology-based recognition of new species in the spider-parasitic genus *Gibellula* (*Hypocreales*, *Cordycipitaceae*) from Thailand. MycoKeys.

[41] Kepler R.M., Sung G.H., Ban S., Nakagiri A., Chen M.J., Huang B., Li Z., Spatafora J.W. (2012). New teleomorph combinations in the entomopathogenic genus *Metacordyceps*. Mycologia.

[42] Spatafora J.W., Sung G.H., Sung J.M., Hywel-Jones N.L., White J.F. (2007). Phylogenetic evidence for an animal pathogen origin of ergot and the grass endophytes. Mol. Ecol..

[43] Kuephadungphan W., Macabeo A.P.G., Luangsa-Ard J.J., Tasanathai K., Thanakitpipattana D., Phongpaichit S., Yuyama K., Stadler M. (2019). Studies on the biologically active secondary metabolites of the new spider parasitic fungus *Gibellula gamsii*. Mycol. Prog..

[44] Kepler R.M., Luangsa-Ard J.J., Hywel-Jones N.L., Quandt C.A., Sung G.H., Rehner S.A., Aime M.C., Henkel T.W., Sanjuan T., Zare R. (2017). A phylogenetically-based nomenclature for *Cordycipitaceae* (*Hypocreales*). IMA Fungus.

[45] Liu Z.L., Wei D.P., Chen J.H., Wu W.J., Liu Z.H., Zhang W., Chen H., Peng X.C., Kang J.C., Qian Y.X. (2025). A new spider-pathogenic species *Gibellula liaoningensis* (*Cordycipitaceae*) from Liaoning Province, China. Phytotaxa.

[46] Johnson D., Sung G.H., Hywel-Jones N.L., Luangsa-Ard J.J., Bischoff J.F., Kepler R.M., Spatafora J.W. (2009). Systematics and evolution of the genus *Torrubiella* (*Hypocreales*, *Ascomycota*). Mycol. Res..

[47] Mendes-Pereira T., de Araújo J.P.M., Kloss T.G., Costa-Rezende D.H., de Carvalho D.S., Góes-Neto A. (2023). Disentangling the taxonomy, systematics, and life history of the spider-parasitic fungus *Gibellula* (*Cordycipitaceae*, *Hypocreales*). J. Fungi.

[48] Tan Y.P., Shivas R.G. (2023). Nomenclatural novelties. Index Aust. Fungi.

[49] Mongkolsamrit S., Noisripoom W., Tasanathai K., Khonsanit A., Thanakitpipattana D., Himaman W., Kobmoo N., Luangsa-Ard J.J. (2020). Molecular phylogeny and morphology reveal cryptic species in *Blackwellomyces* and *Cordyceps* (*Cordycipitaceae*) from Thailand. Mycol. Prog..

[50] Chen M.J., Wang T., Lin Y.A.N., Huang B.O. (2021). *Gibellula flava* sp. nov. (*Cordycipitaceae*, *Hypocreales*), a new pathogen of spider from China. Phytotaxa.

[51] Gao R.X. (1981). Description of a parasitic fungus *Gibellula suffulta* on spiders in Fujian. Acta Microbiol. Sin..

[52] Tzean S.S., Hsieh L.S., Wu W.J. (1998). *Torrubiella dimorpha*, a new species of spider parasite from Taiwan. Mycol. Res..

[53] Kuephadungphan W., Phongpaichit S., Luangsa-Ard J.J., Rukachaisirikul V. (2013). Antimicrobial activity of invertebrate-pathogenic fungi in the genera *Akanthomyces* and *Gibellula*. Mycoscience.

[54] Helaly S.E., Kuephadungphan W., Phainuphong P., Ibrahim M.A.A., Tasanathai K., Mongkolsamrit S., Luangsa-Ard J.J., Phongpaichit S., Rukachaisirikul V., Stadler M. (2019). Pigmentosins from *Gibellula* sp. as antibiofilm agents and a new glycosylated asperfuran from *Cordyceps javanica*. Beilstein J. Org. Chem..

